# A Comparison of Simulation Training and Didactic Teaching Around the Involuntary Detention Process

**DOI:** 10.1192/bjo.2025.10272

**Published:** 2025-06-20

**Authors:** Adam Flynn, Lorraine Darcy, Linda Clarke, Bronagh Sproule, Chris Sharkey

**Affiliations:** Western Health & Social Care Trust, Derry, United Kingdom

## Abstract

**Aims:** Teaching around the involuntary detention process under the Mental Health (Northern Ireland) Order 1986 is typically given to new rotational doctors at changeover. This can include a lot of new and technical information and likely can present as overwhelming. Initially, Quality Improvement Project was commenced to assess whether Forms under the Mental Health Order were being completed correctly pre- and post-traditional changeover teaching session.

In Northern Ireland Form 5s are completed if someone is a voluntary patient who then asks to leave hospital and is found to be a substantial risk to themselves and others. Form 7s are completed if a patient arrives on a detained basis having been assessed by a GP and Approved Social Worker.

We subsequently then developed high-fidelity simulation pilot around a patient presenting with mania and psychosis to begin to compare whether using simulation as a teaching tool was better-retained at 6-week follow-up.

**Methods:** Driver Diagram initially developed to assess areas in which Form 5 and Form 7 detention forms may have errors.

Didactic teaching given at doctor’s changeover in August and December with questionnaires developed to assess pre- and post-understanding.

Subsequent development of high fidelity Simulation using Scottish Sim model around the practicalities of the detention process using a patient with mania and psychosis.

Subsequent follow-up comparison at 6 weeks post-didactic teaching and simulation to compare confidence and retention of information.

**Results:** The trends around completion of Form 5 and Form 7s under Mental Health (Northern Ireland) Order were assessed pre- and post-didactic teaching in July, September and December 2024 was carried out.

Form 5 detention forms in July, September and December had completion rates without errors of 60, 66.6% and 100% respectively.

Form 7 detention forms in July, September and December had completion rates without errors of 35.71%, 50% and 12.5% in July, September and December.

However, in developing pilot Sim we initially ran it with one person in November 2024 and 6 weeks post-simulation, questionnaire resulted in 100% confidence in knowing when to complete Forms appropriately and comment that “simulation has been very useful in completion of forms”.

When we compared this with the didactic teaching in December 2024 this level of confidence around retention of teaching was only 60% (n=3).

**Conclusion:** In reviewing other data such as Systematic Reviews on Simulation in Psychiatry, it is generally seen that information learned is retained better in comparison to didactic teaching.

This would correlate with our own findings, all be it with a small sample size.

As part of my role as Education Fellow, I plan to further develop and expand use of Simulation in the Western Trust to be able to offer more tailored and realistic training around the involuntary detention process as well as other areas in Psychiatry.

This initial data is promising in terms of assessing whether simulation can be used as a more effective teaching tool and something that we plan to roll out more regularly for rotational doctors where resources allow, improving their confidence and scope to deal with the stressful situation of assessing and completing their part of the involuntary detention process under the Mental Health (Northern Ireland) Order 1986.

